# Adsorption Behavior of Gold Ions on Nanofiber Webs Containing Protein Polyhedral Crystals

**DOI:** 10.1021/acsomega.5c01719

**Published:** 2025-05-27

**Authors:** Shuto Matsuura, Takashi Iwahashi, Hajime Mori, Akihiko Tanioka, Hidetoshi Matsumoto

**Affiliations:** † Department of Materials Science and Engineering, Institute of Science Tokyo, 2-12-1 Ookayama, Meguro-ku, Tokyo 152-8552, Japan; ‡ Inspired Micro Crystals, 27-1 Shimouchikawara-cho, Koyama, Kita-ku, Kyoto 603-8132, Japan

## Abstract

Protein polyhedral crystals (PhCs), with well-defined three-dimensional structures and narrow channels/cavities, have the potential to be utilized as stable biobased adsorbents. However, their intrinsic adsorption abilities for metal ions, including precious metals, remain unclear. In this study, protein PhCs were immobilized in polymer nanofiber (PhC@NF) webs via electrospinning with poly­(ethylene-*co*-vinyl alcohol) (EVOH). The morphology and adsorption behavior of the PhC@NF web for precious metal ions such as gold (Au) were investigated by scanning electron microscopy and inductively coupled plasma mass spectrometry, respectively. At 25 °C, Au adsorbed only on PhCs and the PhC@NF web exhibited a maximum Au adsorption capacity of 51.7 mg g^–1^ at pH 1 and a maximum Au adsorption equilibrium constant of 1.02 L mg^–1^ at pH 3, the highest constant among reported Au ion adsorbents. By contrast, Au reduced by PhCs was deposited as particles on EVOH NF web at 65 °C. The study demonstrates that PhCs are promising materials for efficient recovery of Au from low-concentration Au ion solutions.

## Graphical abstract

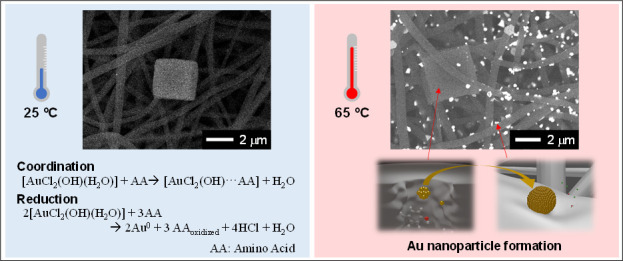

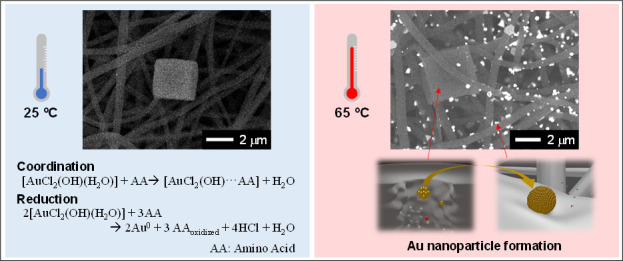

## Introduction

Precious metals such as gold (Au), silver, and platinum play important roles in many industrial fields due to their superior oxidation and corrosion resistance^1,2^ and high electrical conductivity.^3^ In particular, precious metals are utilized as key components in electronic and medical devices. For example, they are employed in electrical wires and connectors in circuit boards^5^ and as plasmonic nanoparticles in diagnostics and therapy systems.^6^ The consumption of precious metals is steadily increasing with expanded use of these devices. Therefore, “urban mining” is becoming more cost-effective than virgin mining,^7^ and adsorption techniques with high selectivity and capacity have attracted attention in the urban mining field.

Biobased adsorbents represent a new class of adsorbents with inherent environmental friendliness for urban and brine mining purposes.^8^ Among them, protein molecules containing specific amino acid (AA) residues and possessing particular topological features exhibit specific affinities for metal ions.^9–11^ Recently, protein crystals have attracted attention among researchers as biobased stable cages with internal pores that can interact with guest molecules/ions via their unique three-dimensional coordination.^12^ Ueno et al. reported Au subnanocluster nucleation within a crystalline protein (ferritin) cage using synchrotron X-ray diffraction.^13^ Notably, cypovirus has been extensively characterized as microscale polyhedra crystals (PhCs) consisting of polyhedrin protein.^14–16^ Many cypovirus particles are embedded in highly stable and sealed PhCs, protecting them from environmental damage. Cypovirus PhCs remain stable even under harsh conditions, such as acidic environments or exposure to chemical reagents like ethanol, 8 M urea, or 10% SDS.^16^ Moreover, cypovirus PhCs function as versatile protein microcrystal carriers of cell growth factors^17,18^ and enable sustained release of cargo proteins.^19^ Although the narrow channels/cavities within cypovirus PhCs can interact with heavy atoms, including precious metals,^16^ their metal adsorption behaviors have not yet been clarified.

In this study, we immobilized cypovirus PhCs in polymer nanofiber (NF) webs composed of poly­(ethylene-*co*-vinyl alcohol) (EVOH) to facilitate manipulation of these tiny protein crystals, and we investigated their Au ion adsorption behavior. Due to its stability, biocompatibility, and hydrophilicity, EVOH is widely used for biomedical applications.^20,21^ EVOH was dissolved in a mixture of water and *n*-propanol to enable electrospinning of cypovirus PhCs, since the PhCs can only be dispersed in water or alcohols with carbon chains containing three carbons. This study aims to clarify the intrinsic adsorption ability of cypovirus PhCs immobilized on a polymer substrate for Au ionic species. To the best of our knowledge, this study is the first to report the quantitative Au ion adsorption behavior of cypovirus PhCs.

## Experimental Section

### Materials

PhCs from Bombyx mori cypovirus (PODS Empty) were purchased from Cell Guidance Systems (Cambridge, UK). EVOH solution (17 wt % in a water (H_2_O)/*n*-propanol (NPA) mixture) (1:1 wt/wt) (Soarnol 16DX) was provided by Nippon Synthetic Chemical Industry (Osaka, Japan). NPA (purity ≥ 99.5%), hydrogen tetrachloroaurate (III) tetrahydrate (HAuCl_4_·4H_2_O, purity ≥ 99.9%), manganese­(II) chloride tetrahydrate (MnCl_2_·4H_2_O, purity ≥ 99.9%), zinc chloride (ZnCl_2_, purity ≥ 99.9%), copper­(II) chloride dihydrate (CuCl_2_·2H_2_O, purity ≥ 99.9%), hydrochloric acid (HCl; 1 M) for volumetric analysis, Au standard solution (1000 mg L^–1^ including 4–6 wt % HCl) for ICP analysis, thiourea (H_2_NCSNH_2_, purity ≥ 98%), iron­(III) chloride hexahydrate (FeCl_3_·6H_2_O, purity ≥ 99%), ammonium thiocyanate (NH_4_SCN, purity ≥ 98%), and sodium hydroxide (NaOH; 0.1 M) for volumetric analysis were purchased from Fujifilm Wako (Osaka, Japan. Ultrapure water (H_2_O) was prepared using a water purification system (Milli-Q Reference; Merck, Darmstadt, Germany). Before use, the PhCs were dispersed in H_2_O. The aqueous dispersions were centrifuged at 12,000*g* and the solvent was exchanged with H_2_O after removing the supernatant. This treatment was repeated three times, and the suspensions were then vacuum-dried at 25 °C for 3 h to obtain purified PhCs (Scheme S1, Supporting Information). Other reagents were used as received without further purification.

### Electrospinning

The preparation of PhC immobilized EVOH nanofiber (PhC@NF) webs is shown in Scheme S1, Supporting Information. First, a 12 wt % EVOH/H_2_O-NPA solution was prepared by diluting the as-provided EVOH solution with H_2_O/NPA mixture (1:1 wt/wt). The purified PhCs were added to the 12 wt % EVOH solution and stirred for 5 min, producing a PhC/EVOH dispersion, which was immediately utilized in electrospinning. The electrospinning apparatus is shown in Figure S1, Supporting Information. The PhC/EVOH dispersion was placed in a plastic syringe with a stainless-steel nozzle (inner diameter, 0.81 mm) as the spinneret. The nozzle was connected to a high-voltage direct current power supply (HAR-100P0.3; Matsusada Precision, Shiga, Japan). Aluminum foil was used as the collector, i.e., as the counter electrode. A constant-volume flow rate was maintained using a syringe infusion pump (MCIP-III; Minato Concept, Tokyo, Japan). The applied voltage was 17 kV, the distance between the nozzle tip and collector was 250 mm, and the flow rate was 5 μL min^–1^. Electrospinning was conducted at 25 °C and <40% relative humidity. Freestanding NF webs were obtained after electrospinning for 1 h. To remove the residual solvent prior to characterization, the prepared samples were vacuum-dried at 25 °C for 3 h.

### Characterization

The surface morphologies of the purified PhCs and PhC@NF webs were observed by scanning electron microscopy (SEM) (JCM-7000; JEOL Ltd., Tokyo, Japan) operated at 5–15 kV. All samples were sputter-coated with Pt. The size distribution of the PhCs and fiber diameter distribution of the NF webs were determined through SEM image analysis of 1000 PhCs and fibers, respectively, using ImageJ software (National Institutes of Health, Bethesda, MD, USA).

The PhC/EVOH interface structure of the PhC@NF web was observed by field-emission scanning electron microscopy (FE-SEM) (SU9000; Hitachi High-Tech Corporation, Tokyo, Japan) in the scanning transmission electron microscopy (STEM) mode operated at 30 kV. The samples were embedded in light-curable resin (Aronix LCR-D800; Toagosei Co., Ltd., Tokyo, Japan) and sectioned to a thickness of approximately 80 nm at −65 °C using an ultramicrotome (Leica UC7; Leica Microsystems GmbH, Wetzlar, Germany). The ultrathin sections were transferred to colloid-coated copper grids for analysis.

Thermogravimetric analysis (TGA) of the purified PhCs was performed using a Thermo plus EVO TG 8120 thermal analyzer (Rigaku, Tokyo, Japan) under a N_2_ atmosphere, heating from 25 to 500 °C at a rate of 10 °C min^–1^. Differential scanning calorimetry (DSC) was performed using a DSC7000X calorimeter (Hitachi High-Tech Corporation) under an N_2_ atmosphere, heating from 0 to 200 °C at a rate of 1 °C min^–1^.

### Adsorption Experiments

PhC@NF web (0.149–3.89 mg) was immersed in aqueous HAuCl_4_·4H_2_O solutions (0.999 g) at various concentrations and pH values. Adsorption experiments were performed in poly­(propylene) (PP) vials with stirring at 120 rpm using a reciprocal shaker for 3 d at 25 °C, 45 °C, and 65 °C under dark conditions. The pH of the adsorbate solutions was adjusted with HCl (Table S1, Supporting Information). The experiments were performed in a constant temperature chamber (LHU-113; ESPEC Corporation, Osaka, Japan). For comparison, NF web without PhCs (EVOH NF, 0.950 mg) was immersed in aqueous HAuCl_4_·4H_2_O solution (1.00 g) at pH 3, and adsorption experiments were performed in PP vials with stirring at 120 rpm using a reciprocal shaker for 3 d at 25 and 65 °C under dark conditions. The adsorption experiments conducted at 25 and 65 °C were performed with adsorbate solutions containing 100 and 200 mg L^–1^, respectively. The Au adsorption capacity was estimated from the amount of Au remaining in the adsorbate solution, which was determined by inductively coupled plasma mass spectrometry (ICP–MS) (7700× ICP–MS; Agilent Technologies, Santa Clara, CA, USA). Prior to analysis, the adsorbate solutions were filtered through 0.2 μm poly­(tetrafluoroethylene) (PTFE) membranes (Kanto Chemical, Tokyo, Japan). The amount of adsorbed Au per unit mass of PhC@NF web (*q* [mg g^–1^]) was calculated as follows

1 $$q=\frac{\left(C_{0}-C\right)V}{m}$$

where *C*_0_ and *C* [mg L^–1^] are the concentrations of adsorbate solution before and after adsorption, respectively; *V* [L] is the volume of solution; and *m* [g] is the weight of PhC@NF web measured using an ultramicrobalance (XP2U; Mettler Toledo, Greifensee, Switzerland).

For adsorption kinetics analysis, PhC@NF web (30 mg) was immersed in aqueous HAuCl_4_·4H_2_O solution (10 g) containing 100 mg L^–1^ Au at pH 3. Adsorption experiments were performed in PP vials with stirring at 120 rpm using a magnetic stirrer under dark conditions. An aliquot of 10 mg was sampled at each time point, the 1000-fold diluted sample was filtered through a PTFE membrane, and the Au concentration was subsequently analyzed by ICP–MS. The decrease in the Au adsorption rate due to the sampling volume was considered to be negligible.

The elemental composition of the PhC@NF webs after Au adsorption was determined by energy dispersive spectroscopy (EDS) (AMETEK/EDAX Genesis; EDAX Inc., Mahwah, NJ, USA) combined with FE-SEM (SU9000; Hitachi High-Tech Corporation) operated at 10 kV. Samples were coated with osmium prior to analysis. Surface-sensitive analysis of the oxidation states of Au-adsorbed on the PhCs was performed via X-ray photoelectron spectroscopy (XPS) (PHI VersaProbe III; Ulvac PHI, Kanagawa, Japan) equipped with a monochromatic Al Kα radiation source (25 W, 15 kV). The acquired spectra were charge-corrected using the C 1s and Au 4f signals at 285 and 84 eV, respectively. The X-ray spot size was set to a diameter of 100 μm. The color of the NF webs before and after Au adsorption was observed using a USB output microscope (L-KIT501; Hozan, Osaka, Japan) with Cam Plus software (Hozan).

### Desorption Experiments

Au-adsorbed PhC@NF web (3 mg, obtained from the adsorption experiment conducted with 200 mg L^–1^ Au adsorbate solution at pH 3 and 25 °C ± 1 °C for 3 d) was immersed in eluent solution (10 g, mixture of 5 mM H_2_NCSNH_2_, 10 mM FeCl_3_·6H_2_O, and 5 mM NH_4_SCN adjusted to pH 2 using HCl). Desorption experiments were performed for 1 d in PP vials with stirring at 120 rpm using a reciprocal shaker at 25 °C under an N_2_ atmosphere and dark conditions.^22^ The Au desorption capacity was estimated from the amount of Au present in the eluent solution, which was determined by inductively coupled plasma optical emission spectrometry (ICP-OES) (5100 VDV; Agilent Technologies, Tokyo, Japan). The elemental composition of the Au-desorbed PhC@NF web was determined by EDS.

### Isotherm, Kinetic, and Thermodynamics Equations

For the adsorption isotherms of Au on the PhC@NF web, the equilibrium Au adsorption capacity (*q*_e_ [mg g^–1^]) was fitted against the equilibrium Au concentration (*C*_e_ [mg L^–1^]) using the Langmuir model,^23^ as follows

2 $$q_{e}=\frac{q_{\max}K_{L}C_{e}}{1+K_{L}C_{e}}$$

where *q*_max_ [mg g^–1^] is the maximum adsorption capacity, and *K*_L_ [L mg^–1^] is the Langmuir adsorption equilibrium constant.

For the adsorption kinetics of the PhC@NF web, the amount of adsorbed Au per unit mass of PhC@NF web (*q*_*t*_ [mg g^–1^]) was fitted against time (*t* [h]) using the following pseudo-first-order model (eq 3),^24^ pseudo-second-order model^25^ (eq 4), and intraparticle diffusion model^26^ (eq 5)­

3 $$q_{t}=q_{e}\left(1-e^{-k_{1}t}\right)$$

where *q*_e_ [mg g^–1^] is the equilibrium adsorption capacity, and *k*_1_ [h^–1^] is the pseudo-first-order rate constant.

4 $$q_{t}=\frac{{q_{e}}^{2}k_{2}t}{1+q_{e}k_{2}t}$$

where *k*_2_ [g (mg h)^−1^] is the pseudo-second-order rate constant.

5 $$q_{t}=k_{id}t^{\frac{1}{2}}+C$$

where *k*_id_ [mg (g h^1/2^) ^–1^] is the intraparticle diffusion rate constant, and *C* [mg g^–1^] is the intercept under different adsorption processes.

### Density Functional Theory (DFT) Calculations

DFT calculations were performed using the Gaussian 16 program^27^ with the wB97XD hybrid functional.^28,29^ The optimized geometries and energies of all structures were obtained using the Los Alamos National Laboratory 2-double-ζ (LANL2DZ) basis set^10,30^ for Au atoms and the default two triple-ζ plus polarization (def2TZVP) basis set^10,31^ for the other atoms. We adopted the integral equation formalism polarizable continuum model using water as the solvent to incorporate the bulk effect of hydration on the structures and energies.^10,32^

The interaction energies of Au­(III) species with AA residues were calculated as previously described.^10^ The interaction energy of [AuCl_4_]^−^ was deduced by assuming equilibrium conditions with AAs with (formula [Disp-formula eq6]) and without (formula [Disp-formula eq7]) deprotonation, as shown below

6 $${\left[{AuCl}_{4}\right]}^{-}+AA+4H_{2}O\to \left[AA\cdot {AuCl}_{3}\right]+\left[3H_{2}O\cdot H_{3}O^{+}\cdot {Cl}^{-}\right]$$

7 $${\left[{AuCl}_{4}\right]}^{-}+AA+4H_{2}O\to \left[AA\cdot {AuCl}_{3}\right]+\left[4H_{2}O\cdot {Cl}^{-}\right]$$

The interaction energy of [AuCl_2_(OH)­(H_2_O)] was determined based on possible interactions between the Au complex and AAs via Au atoms (coordination interaction, i.e., formula [Disp-formula eq8]) and OH (hydrogen bonding, i.e., formula [Disp-formula eq9]), as shown below

$$\left[AuCl_{2}\left(OH\right)\left(H_{2}O\right)\right]+AA+4H_{2}O\to \left[AA\cdot AuCl_{2}\left(OH\right)\right]+\left[4H_{2}O\cdot H_{3}O^{+}\right]\;\text{or}\;\;5H_{2}O$$ 8

9 $$\left[AuCl_{2}\left(OH\right)\left(H_{2}O\right)\right]+AA+4H_{2}O\to \left[AuCl_{2}\left(OH\right)\cdot \cdot \cdot AA\right]+\left[4H_{2}O\cdot H_{3}O^{+}\right]\;\text{or}\;5H_{2}O$$

where “···” denotes hydrogen bonding. The interaction energies of Au­(III) species with the peptide/protein backbone and N/C-terminal AAs were calculated in the same manner.^10^

## Results and Discussion

### Immobilization of PhCs in the NF Web

Figure 1 shows an SEM image and the size distribution of the purified PhCs, revealing microscale cubic shapes with side lengths of 2.58 ± 0.98 μm. Figure 2a and b show surface SEM images and the fiber diameter distributions of the prepared NF webs with and without PhCs, respectively. The use of 12 wt % EVOH solution during electrospinning resulted in smooth and bead-free NFs. The average fiber diameter (*D*) of the NF webs was approximately 560 nm, and the immobilization of PhCs within the NF web did not cause a significant diameter change. Within the NF network, the PhCs were partially coated with an EVOH layer and stably immobilized (Figure 2c). In the ultrathin section of the web, the dark gray cube of a single PhC is covered with an approximately 150–250 nm thick EVOH layer (the light gray area at the top and left of the cube in Figure 2d). The solutes in the electrospinning solution (PhC/EVOH/solvent = 1.8/11.8/86.4 in weight) were almost completely collected by the PhC@NF web. Therefore, the PhC/EVOH composition of the PhC@NF web was considered to be almost same as that of the spinning solution (PhC/EVOH = 0.15 in weight). The obtained freestanding NF webs, with a typical thickness of approximately 30 μm (Figure 2e), were utilized in the adsorption experiments.

**Figure 1:**
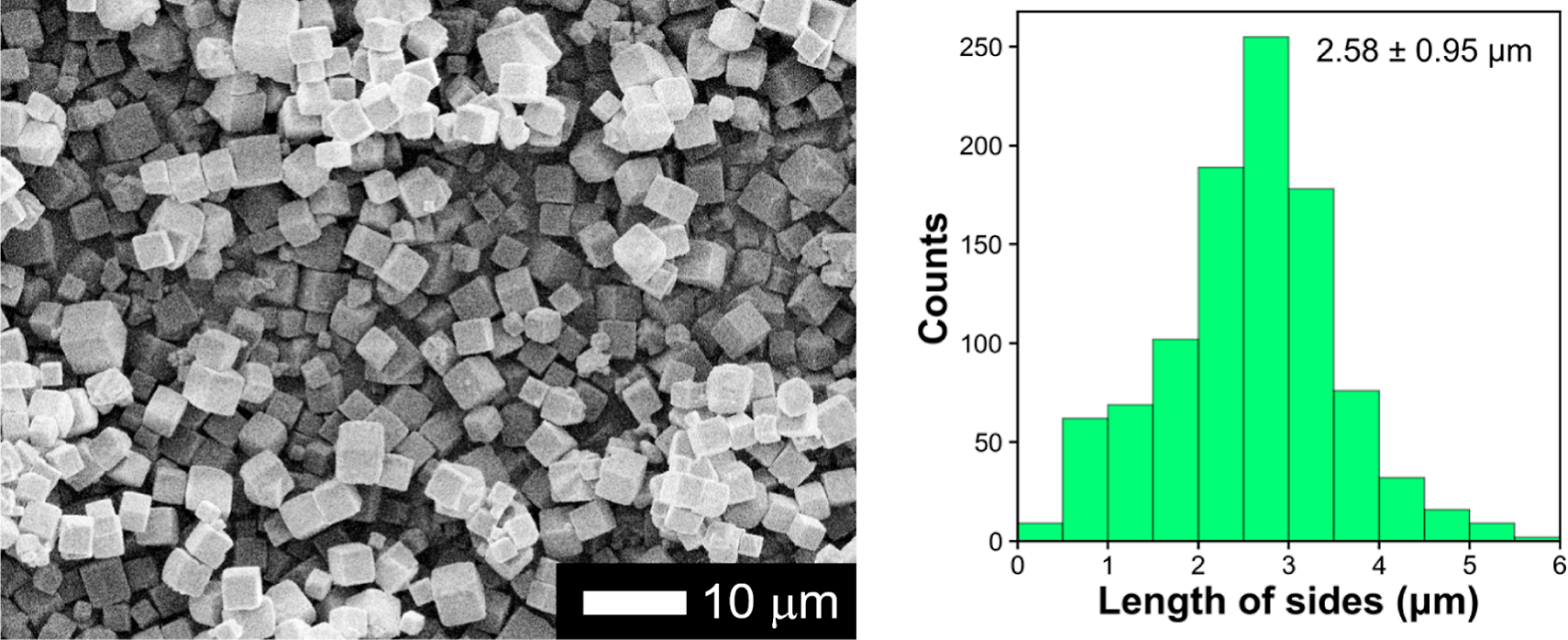
SEM image and size distribution of purified PhCs.

**Figure 2:**
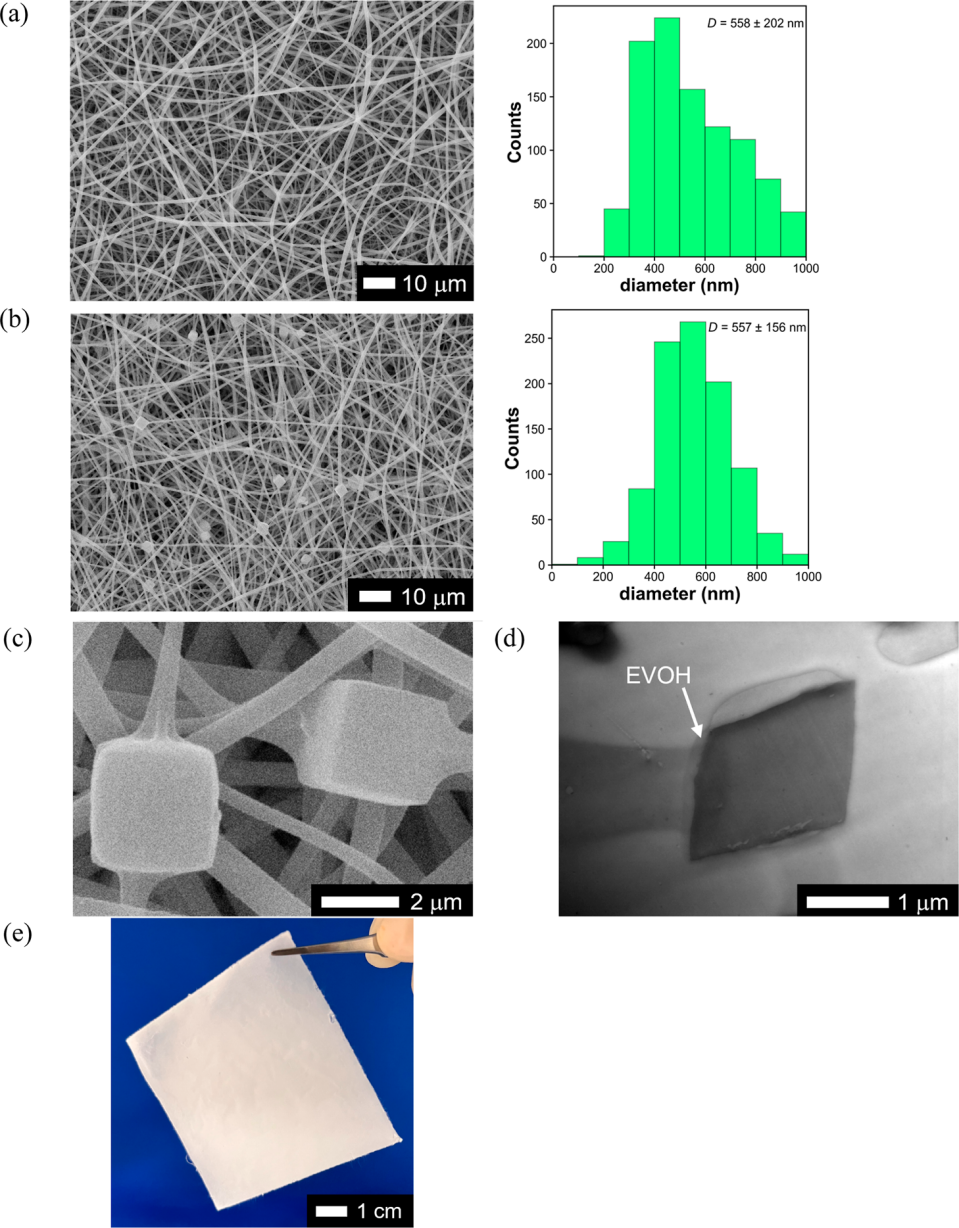
Surface SEM images and fiber diameter distribution of the prepared NF webs: (a) NF web without PhCs (EVOH NF) and (b) PhC@NF web. (c) High-magnification surface SEM image of PhC@NF web. (d) Bright-field STEM image of a typical immobilized PhC within the PhC@NF web. (e) Photograph of a typical PhC@NF web.

### Au Adsorption Behavior of the PhC@NF Web

Aqueous HAuCl_4_·4H_2_O solution was adopted as the precious metal ion adsorbate solution because the obtained PhC@NF webs exhibited excellent Au ion adsorption characteristics (Figure S2, Supporting Information). Figure 3 shows a typical SEM image of the Au-adsorbed PhC@NF web immersed in aqueous HAuCl_4_·4H_2_O solution and the corresponding EDS analysis. Notably, the fiber and PhC structures were maintained after the adsorption experiment (Figure 3a). Additionally, the adsorbed Au was clearly localized in the PhC region of the PhC@NF web (Figure 3b–d).

**Figure 3:**
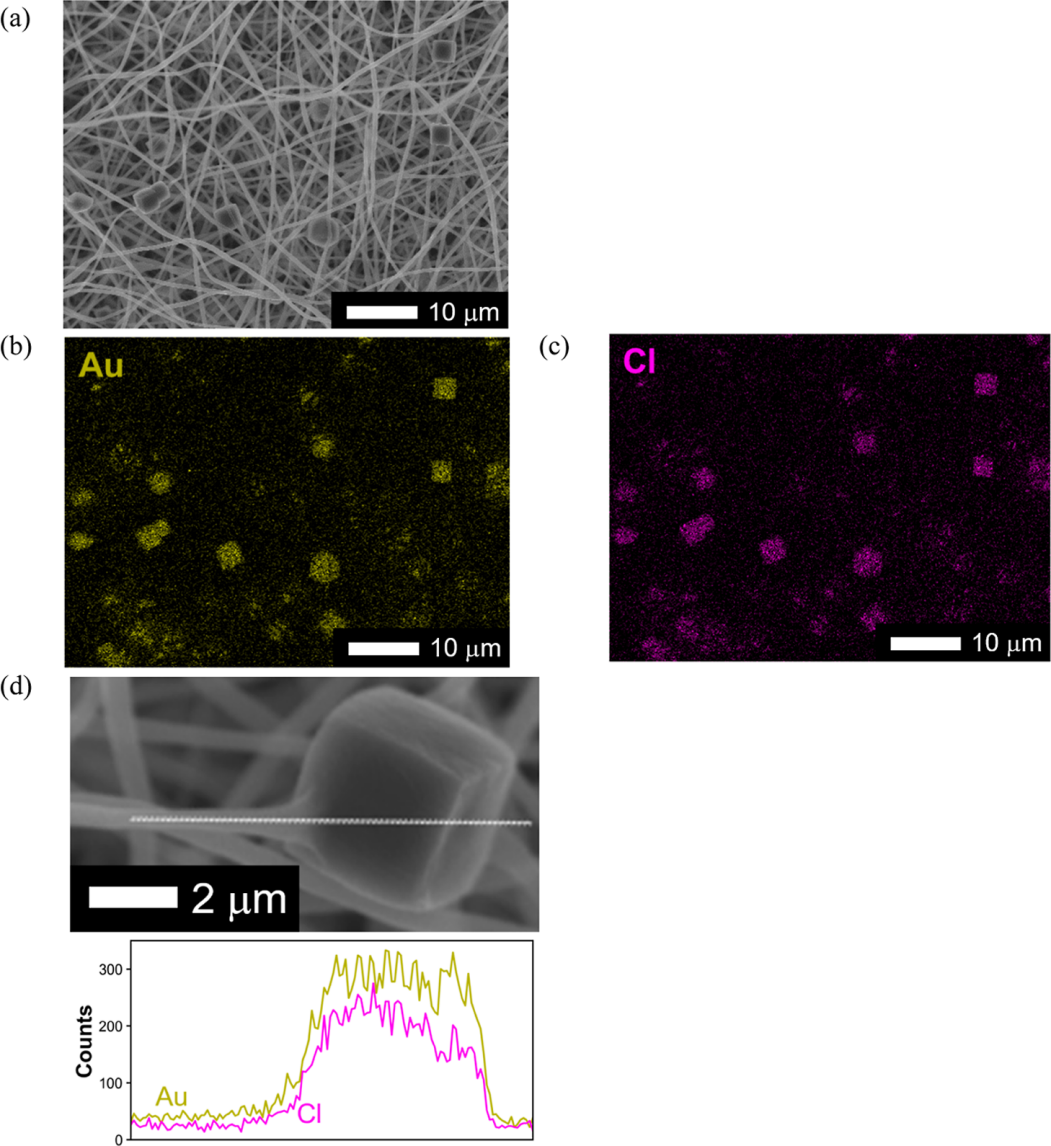
(a) Typical surface SEM image of the PhC@NF web after the Au adsorption experiment in 100 mg L^–1^ Au ion solution at pH 3 and 25 °C. Corresponding EDS mapping of (b) Au-M and (c) Cl-K. (d) Corresponding EDS line analysis near a single PhC immobilized within the PhC@NF web.

We considered PhCs as a simple adsorbent and analyzed the obtained adsorption isotherms using the Langmuir model for simplification, since Au adsorption occurred only within PhCs in the webs. Figure 4a–c shows the plotted results, which were well fitted by the simple Langmuir model (eq 2). The isotherm adsorption parameters *q*_max_ and *K*_L_, obtained by nonlinear regression analysis of the adsorption isotherms, are listed in Table 1. The adsorption isotherms of Au exhibited pH-dependent behavior: the *q*_max_ value decreased with increasing pH, while the *K*_L_ value increased with increasing pH. The highest *q*_max_ and *K*_L_ values were obtained at pH 1 and 3, respectively. By contrast, the NF web without PhCs (EVOH-NF) exhibited no Au adsorption capacity (Figure 4c, blue circle), which was consistent with the EDS results shown in Figure 3b–d. These results suggested that the intrinsic adsorption ability of PhCs for Au ionic species could be estimated by the amount of immobilized PhCs within the PhC@NF web (i.e., the PhC/EVOH composition described above). The highest *q*_max_ value (51.7 mg g^–1^, equivalent to 392 mg g^–1^ per unit weight of PhC) was relatively low compared to those of previously reported Au adsorbents under similar conditions (Table S2, Supporting Information); however, the highest *K*_L_ value (1.02 L mg^–1^) was the highest value among reported Au ion adsorbents.^33–38^ This finding reflects the intrinsic microstructural features of PhCs, including densely packed proteins and narrow channels/cavities.^16^

**Figure 4:**
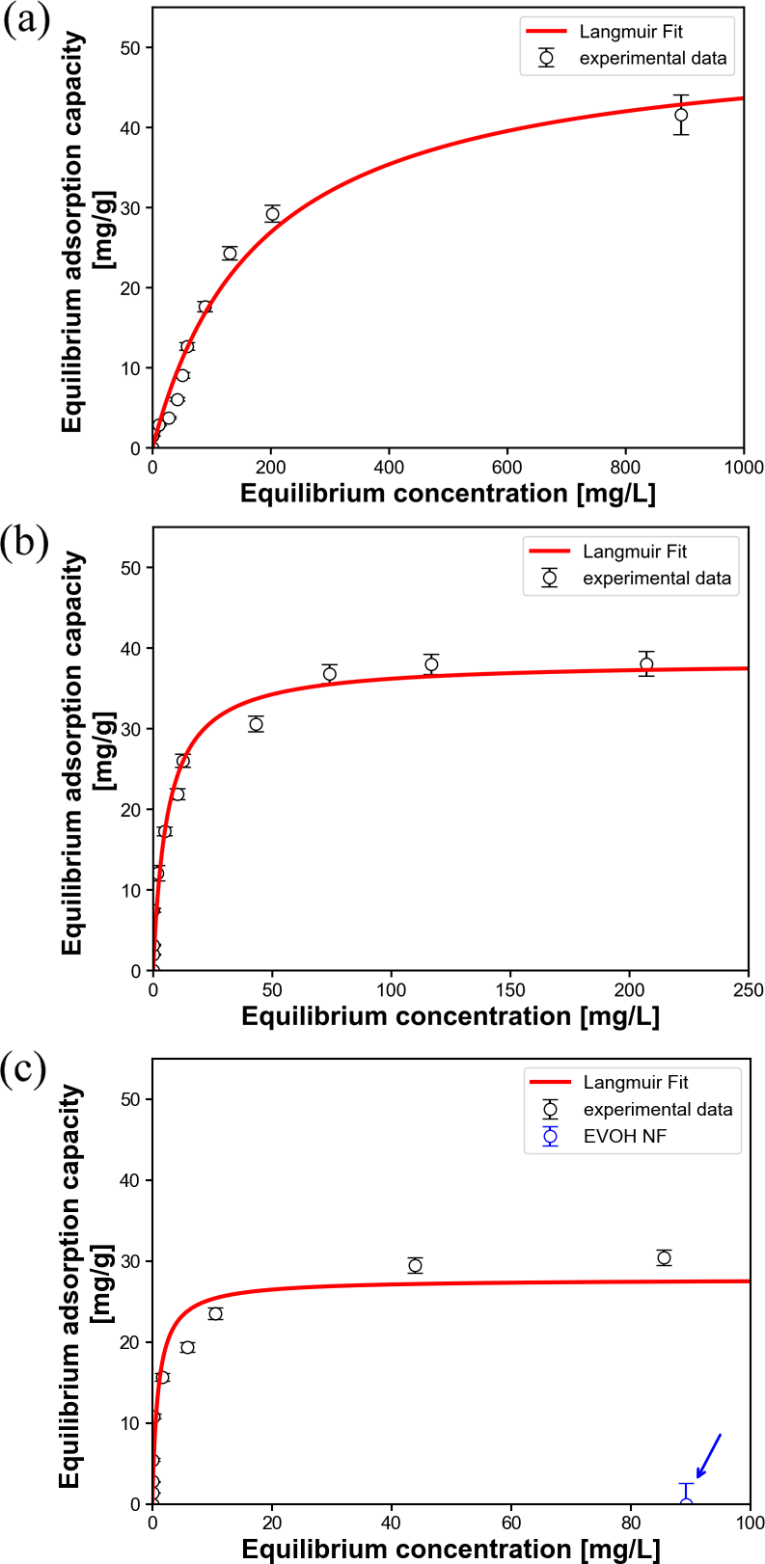
Adsorption isotherms of Au on the PhC@NF web at 25 °C and (a) pH 1, (b) pH 2, and (c) pH 3. The blue circle indicates the Au adsorption capacity of the NF web without PhCs (EVOH NF). The solid lines correspond to theoretical fitting using the Langmuir model. The characteristic parameters are listed in Table 1.

**Table 1:** Isotherm Adsorption Parameters of Au Ions on the PhC@NF Web at Various pH Values, Derived from the Langmuir Model

pH	*q*_max_ [mg g^–1^]	*K*_L_ [L mg^–1^]	*R* ^2^
1	51.7	0.00544	0.976
2	38.4	0.167	0.961
3	27.8	1.02	0.903

The pH-dependent Au adsorption behavior was attributed to the presence of stable Au species in the adsorbate solution and the charge (protonation) state of AA residues within the PhCs at a given pH. The estimated pH dependence of the fraction of trivalent Au ionic species in the adsorbate solution and the p*K*_a_ values of AA side chains within the PhCs are shown in Figure S3 and Table S3, Supporting Information, respectively.^39,40^ For example, the fraction of [AuCl_4_]^−^ decreased as the pH of the adsorbate solution increased from 1 to 3, while the proportion of [AuCl_2_(OH)­(H_2_O)] increased.^39^

However, understanding the pH-dependent Au adsorption behavior of the PhC@NF web remained challenging. Therefore, XPS measurements were performed to detect the Au species adsorbed on the PhCs, and the interaction energies between the Au species and AA residues within the PhCs were estimated by simplified DFT calculations (described in a later section). Figure 5 and Table 2 show the Au 4f XPS spectra and peak analysis of the Au-adsorbed PhCs at various pH values. XPS spectra over the entire measured energy range are shown in Figure S4, Supporting Information. The XPS results showed that the PhC surfaces contained trivalent and elemental Au species (Au­(III) and Au(0), respectively) under all pH conditions, indicating that some AA residues within the PhCs reduced trivalent Au species. Inside the PhC, tyrosine (Tyr) residues, which can interact with Au­(III) ions and reduce them to Au(0), form a cluster of Tyr residues.^16^ One possibility is that the Au­(III) ions are selectively incorporated into the Tyr cluster, where they are reduced to Au(0) and retained via chemical interactions such as Au–OH.^41^ Notably, the XPS data were obtained from the surface region of the PhCs and not the entire PhCs. Therefore, a more detailed discussion of the Au adsorption isotherm is difficult at present. For a more accurate analysis of the Au­(III) and Au(0) adsorption isotherms, a quantitative analysis of the adsorbed Au species on the whole PhCs is strongly required.

**Figure 5:**
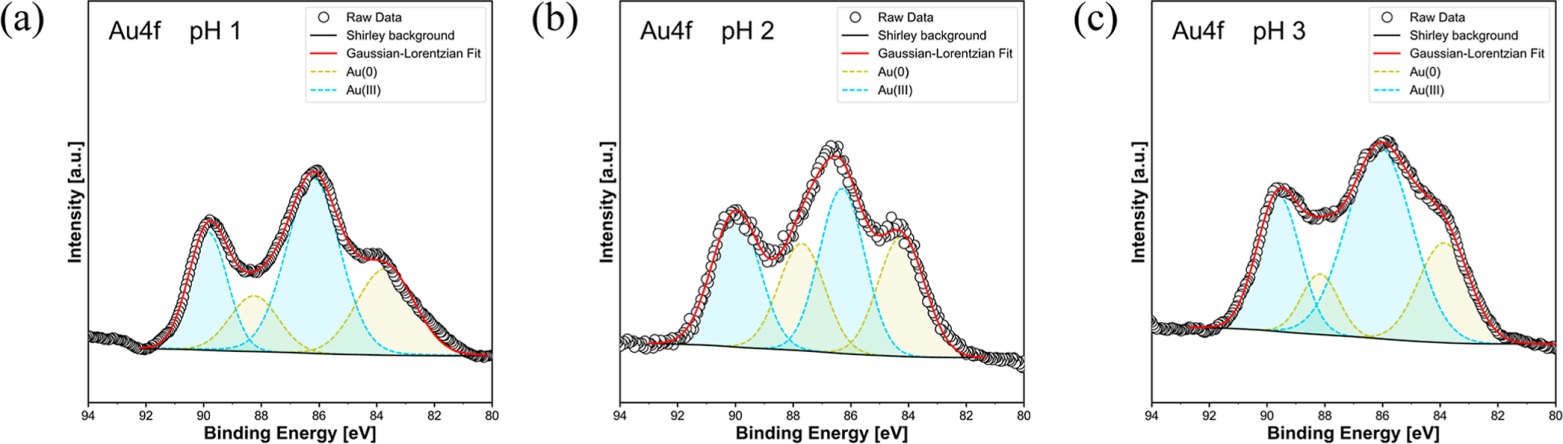
Au 4f XPS spectra of Au-adsorbed PhCs with maximum adsorption capacity at (a) pH 1, (b) pH 2, and (c) pH 3.

**Table 2:** XPS Analysis of Au-Adsorbed PhCs at Different pH Values

pH	Au(0) [%]^a^	Au(I) [%]^a^	Au(III) [%]^a^
1	35	0	65
2	43	0	57
3	28	0	72

a Atomic %.

Au adsorption depends on the mass transfer of Au species from the bulk solution to the binding sites on the surface of or within the solid adsorbents. The time-dependent Au adsorption data were analyzed using three kinetic models (eqs 3–5) and were well fitted by the intraparticle diffusion model, as shown in Figure 6a,b (*R* ^2^ values are listed in Table 3),^26^ suggesting that the kinetic process consisted of two steps: rapid bulk diffusion (step 1) and slow internal diffusion (step 2). In particular, step 2 continued for >2 d and was the dominant step for Au adsorption on the PhC@NF web. This result was consistent with the PhCs having narrow channels/cavities.^16^ Since, Au adsorption occurred only within the PhCs in the web, both the electrostatic adsorption of Au­(III) by basic AA residues and reduction to Au(0) by Tyr residues were included in the step 2. In addition, the PhCs were partially covered by a thin, semicrystalline EVOH layer (Figures 2d and 3d). Incomplete coverage by the EVOH layer and a low degree of crystallinity in the NFs of approximately 27% (obtained from DSC measurements, Figure S5, Supporting Information) did not prevent the diffusion of Au species through the thin layer.

**Figure 6:**
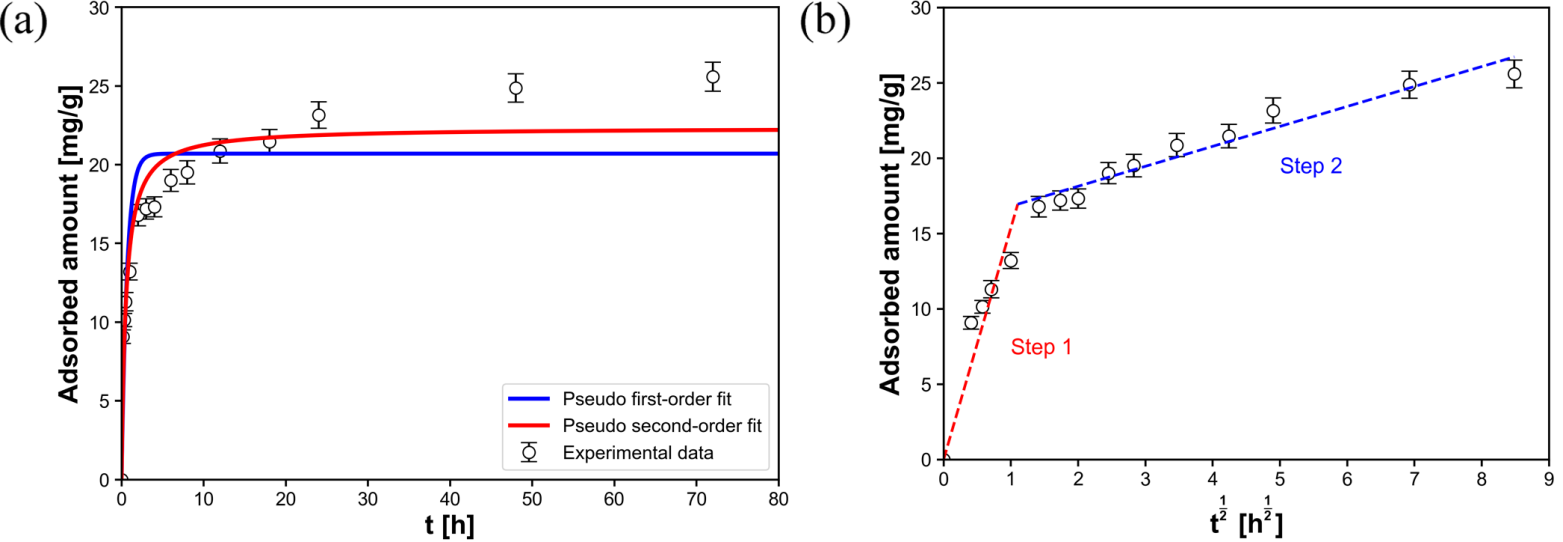
Time-dependent Au adsorption on the PhC@NF web at 25 °C and pH 3 and theoretical fitting based on (a) pseudo first-order and pseudo second-order models and (b) the intraparticle diffusion model. The characteristic parameters are listed in Table 3. Three samples were measured for each condition, and the mean value (±standard deviation) is indicated.

**Table 3:** Kinetic Parameters of Au Ion Adsorption on the PhC@NF Web, Derived from Three Kinetic Models

pseudo first order			pseudo second order			intraparticle diffusion		
*q*_e_ [mg g^–1^]	*k*_1_ [h^–1^]	*R* ^2^	*q*_e_ [mg g^–1^]	*k*_2_ [h^–1^]	*R* ^2^	*k*_id,step1_ [mg (g h^1/2^) ^–1^]	*k*_id,step2_ [mg (g h^1/2^) ^–1^]	*R* ^2^
20.7	1.50	0.826	22.4	0.0853	0.919	15	1.3	0.993

Evaluating the adsorbed Au recovery performance from the Au-adsorbed PhC@NF web and its selective Au adsorption capacity was of great importance for practical applications. The results of the desorption experiments indicated that the adsorption/desorption reaction of PhCs was stoichiometrically reversible (Figure 7a). Moreover, the fiber and PhC structures were maintained after the desorption experiment, and the adsorbed Au was almost completely removed from the PhC region of the PhC@NF web (Figure S6, Supporting Information). Since the eluent solution contained thiourea (H_2_NCSNH_2_), ferric chloride (FeCl_3_), and ammonium thiocyanate (NH_4_SCN), the desorption reaction probably proceeded as follows: the adsorbed elemental Au was oxidized by Fe­(III) ions to trivalent Au­(III) ions, with thiourea acting as a catalyst.^22^ Subsequently, the oxidized and originally adsorbed Au­(III) ions formed complexes with [SCN]^−^, resulting in their desorption from the PhC@NF web.

**Figure 7:**
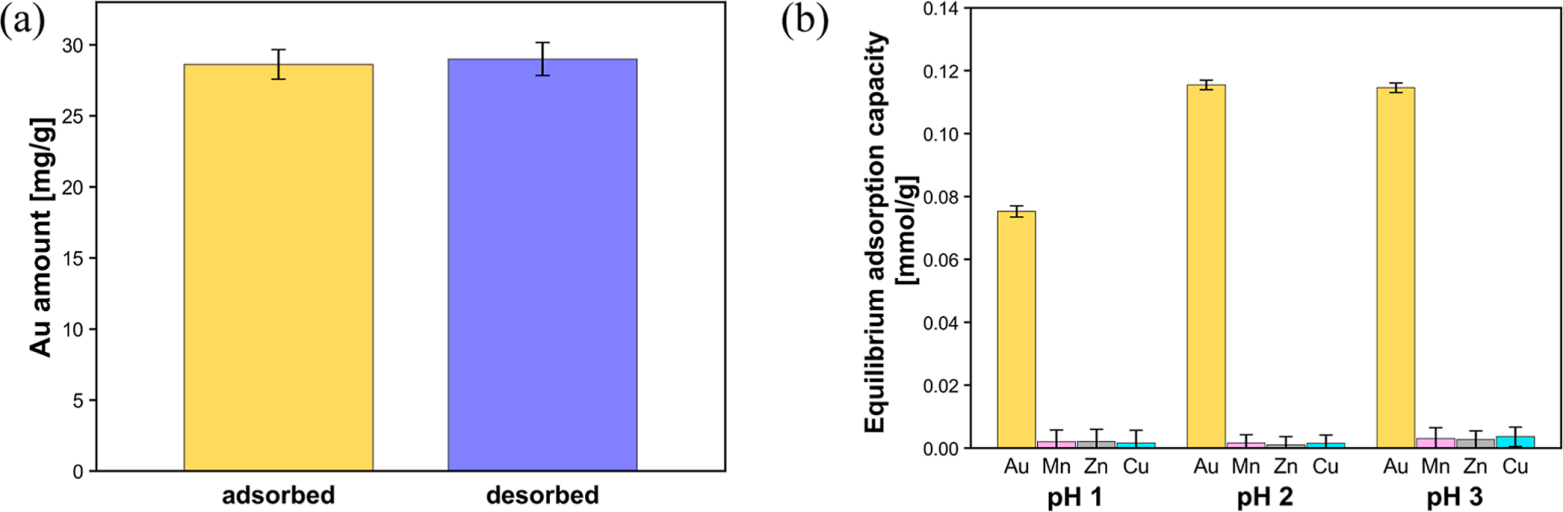
(a) Amounts of Au-adsorbed and desorbed on the PhC@NF web. (b) Equilibrium adsorption capacity of the PhC@NF web in a mixed system containing Au, Mn, Zn, and Cu ions (50 mM). Three samples were measured for each metal, and the mean value (±standard deviation) is indicated.

The intrinsic high *K*_L_ value of the PhCs for Au ion adsorption enabled highly selective Au recovery from solutions with dilute Au ion concentrations. Herein, we focused on “seawater near undersea mineral deposits”^42^ as a potential application for a high *K*_L_ adsorbent, and adsorption experiments were conducted using a model mixed system containing Au ions and other transition metal ions (Mn, Zn, and Cu, which are commonly found in mineral deposits^43,44^ ). The experimental conditions were the same as in the previous batch adsorption experiments, with concentrations of 50 mM for each metal ion. The results showed that the PhC@NF web exhibited 100–350 times higher adsorption capacities for Au species than for the other transition metals (Figure 7b). The higher electronegativity value of 2.54 for Au compared to that of the other transition metals (1.55 for Mn, 1.65 for Zn, 1.9 for Cu) led to unique pH-dependent fractions of trivalent Au ionic species in the adsorbate solution (Figure S3, Supporting Information), with negative [AuCl_4_]^−^ predominantly found at pH 1 and neutral [AuCl_2_(H_2_O)­(OH)] predominantly found at pH 3. By contrast, the other metal ions possessed positive charges in the adsorbate solutions at pH 1–3.

### DFT Calculations

The optimized interaction energies of Au­(III) species with the AA residues were determined by simplified DFT calculations. The role of the AA residues and backbone groups in Au adsorption by the PhCs was investigated in detail, particularly at pH 1 and 3, since the PhCs exhibited the highest *q*_max_ and *K*_L_ values for Au adsorption at pH 1 and 3, respectively. DFT calculations were performed to investigate the interactions and corresponding reaction energies of the Au species (AuCl_3_ for pH 1 and AuCl_2_(OH) for pH 3) with various AAs and backbone groups within the PhCs. Representative optimized structures (cystine) are illustrated in Figure S7, Supporting Information. The optimized structures revealed coordination interactions between AuCl_3_ and AuCl_2_(OH) with all AAs and backbone groups (formulas [Disp-formula eq6]–[Disp-formula eq8]), with their respective reaction energies presented as red circles and green triangles in Figure 8. However, while some AAs and backbone groups exhibited an optimized structure via hydrogen bonding with AuCl_2_(OH) (Formula 9, and noted by orange stars in Figure 8), other AAs exhibited an optimized structure with reduced Au species ([AuCl_2_]^−^) and oxidized AAs, rather than via hydrogen bonding (noted by blue squares in Figure 8). A typical reduction reaction of Au species in this system can be described as follows

10 $$\left[{AuCl}_{2}\left(OH\right)\left(H_{2}O\right)\right]+AA+4H_{2}O\to {\left[{AuCl}_{2}\right]}^{-}+{AA}_{oxidized}+H^{+}+5H_{2}O$$

where AA_oxidized_ is an AA in an oxidized state.

**Figure 8:**
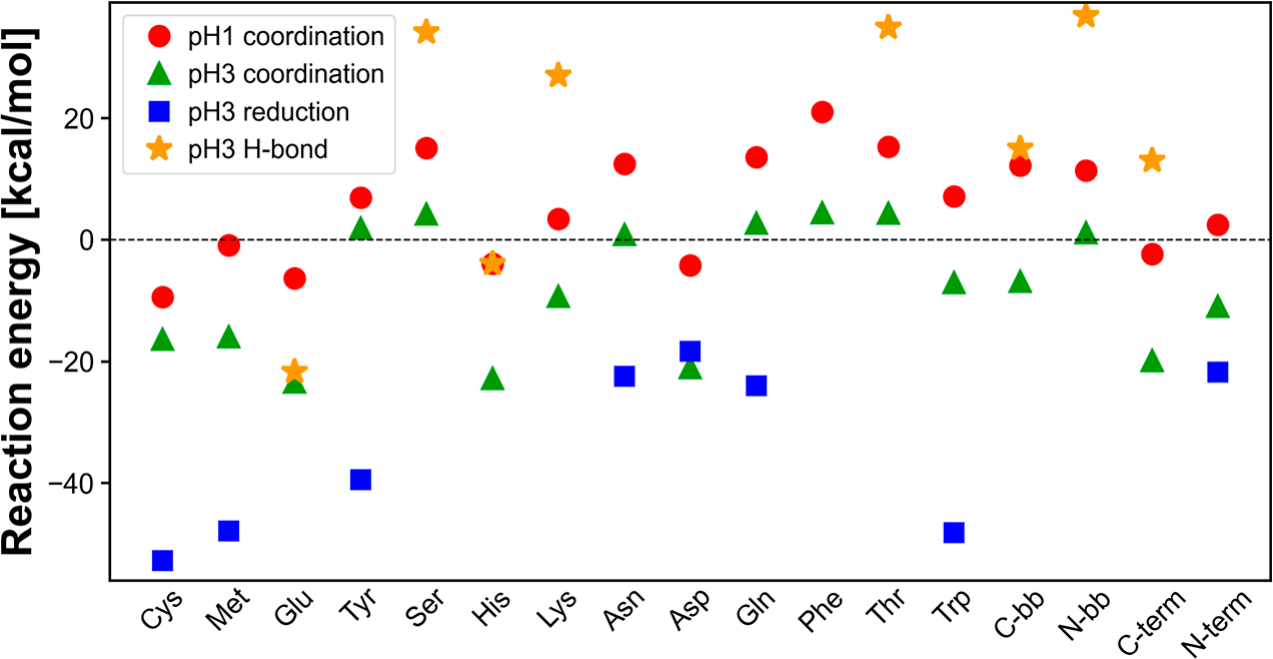
Reaction energies of coordination interactions of Au species, [AuCl_4_]^−^ (red circles) and [AuCl_2_(OH)­(H_2_O)] (green triangles), and energies of hydrogen bonding (orange stars) and reduction reactions (blue squares) of [AuCl_2_(OH)­(H_2_O)] calculated with various AAs and backbone groups.

Comparing the reaction energies of the coordination interactions of [AuCl_4_]^−^ (red circles in Figure 8) and [AuCl_2_(OH)­(H_2_O)] (green bars in Figure 8) showed lower reaction energies for [AuCl_2_(OH)­(H_2_O)] than for [AuCl_4_]^−^ in all optimized structures. This result suggests that [AuCl_2_(OH)­(H_2_O)] generally has higher binding affinities with peptides or proteins than [AuCl_4_]^−^, supporting the higher adsorption coefficient at pH 3 than at pH 1 (Table 1), where [AuCl_2_(OH)­(H_2_O)] was predominantly present (Figure S3, Supporting Information). Furthermore, the reduction reactions (blue squares in Figure 8) exhibited lower reaction energies than the coordination interactions (green triangles in Figure 8) and hydrogen bonding (orange stars in Figure 8), indicating that the reduction reaction from Au­(III) to Au­(I) was energetically favorable with some AA residues. The DFT calculations did not contradict the XPS results showing the presence of elemental Au. The higher adsorption capacity at pH 1 than at pH 3 could not be explained by these results alone, but was possibly due to thermodynamic contributions, such as Donnan equilibrium at the PhC/adsorbate solution interface.^45^ The dominant AA residues within the PhCs, such as arginine (Arg), lysine (Lys) and histidine (His), were mostly positively charged while other AA residues remained neutral under the given conditions^40^ (Table S3, Supporting Information). The positively charged PhC surface attracted negatively charged species such as [AuCl_4_]^−^, resulting in an increased Au concentration near the PhC surface in the equilibrium state.

### Temperature-Dependent Au Adsorption Behavior of the PhC@NF Web

Since the PhC@NF web exhibited the highest *K*_L_ value among the reported Au ion adsorbents at pH 3, it is important to investigate the effect of temperature on the Au adsorption behavior of the PhC@NF web at this pH for basic and applied research purposes. The adsorption isotherms at 25 °C, 45 °C, and 65 °C and pH 3 were obtained as previously described. The TGA curve of the PhCs is shown in Figure S8, Supporting Information, with the weight loss at 65 °C of 0.90%, indicating that the effect of the temperature conditions on the PhC samples is negligible. The obtained isotherms are shown in Figure 9a–c. The equilibrium adsorption capacity (*q*_e_) increased with increasing in temperature. In addition, at 45 and 65 °C, the NF web without PhCs (EVOH NF) exhibited substantial adsorption capacity for Au (Figure 9b,c, blue circle). EVOH polymer with −OH group can reduce Au ions to elemental Au in an aqueous solution, known as polyol process,^46^ and the reduced elemental Au easily form Au clusters inside or on the surface of solid-state EVOH.^47^ Note that the Au adsorption capacity on the PhC@NF web at 45 and 65 °C was significantly higher than the Au adsorption capacity on the EVOH NF.

**Figure 9:**
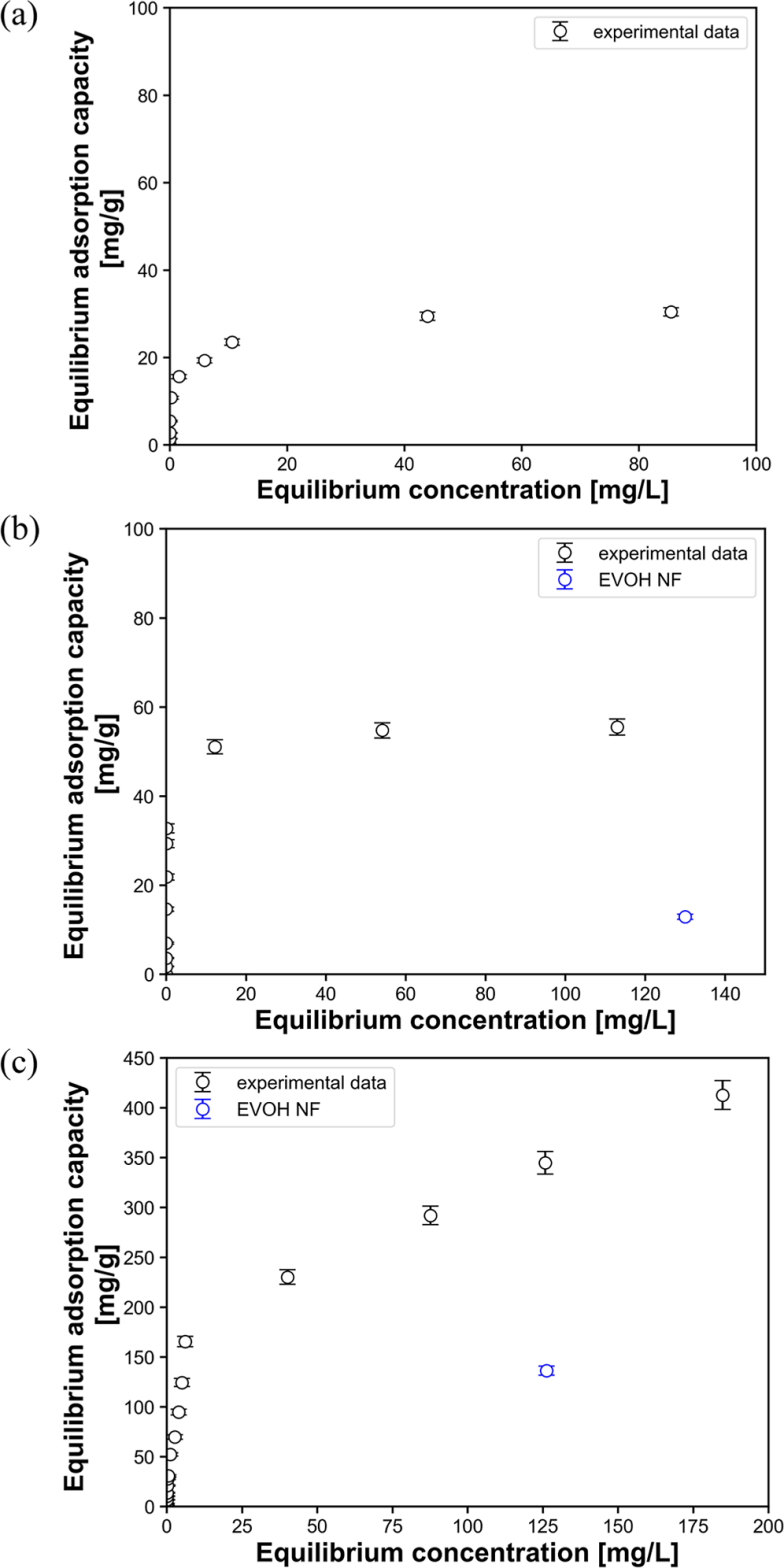
Adsorption isotherms of Au on the PhC@NF web at pH 3 and (a) 25 °C, (b) 45 °C, and (c) 65 °C. The blue circle indicates the Au adsorption capacity of the NF web without PhCs (EVOH NF).

SEM–EDS analysis was performed to clarify the temperature-dependent Au adsorption behavior on the PhC@NF web. Figure 10a–c shows the results of the EDS spot analysis of the Au-adsorbed PhC@NF web at 25 °C, 45 °C, and 65 °C, indicating that the amount of Au adsorbed on the PhCs decreased significantly with increasing temperature. In addition, many nanoparticles (NPs) were deposited on the NF surface (Figure 11a,c) and their size increased as the adsorption proceeded (Figure 11b,d). The EDS line analysis confirmed that the deposited NPs were composed of elemental Au (Figure 11e). Corresponding to the microstructural change, the color of the Au-adsorbed PhC@NF web changed from pink to yellow (Figure S9d,e, Supporting Information), which is consistent with the size-dependent surface plasmon resonance of Au NPs.^48^ For comparison, a photograph and a SEM image of the Au-adsorbed NF web without PhCs (EVOH NF) at 65 °C and pH 3 are shown in Figure S9a,b, Supporting Information, demonstrating the different color and Au NP agglomerate structure compared with the Au-adsorbed PhC@NF webs (Figures 11a,b and S10b–e, Supporting Information). In addition, the EDS spot analysis suggested that the agglomerates on the Au-adsorbed NF web without PhCs were composed of reduced elemental Au.

**Figure 10:**
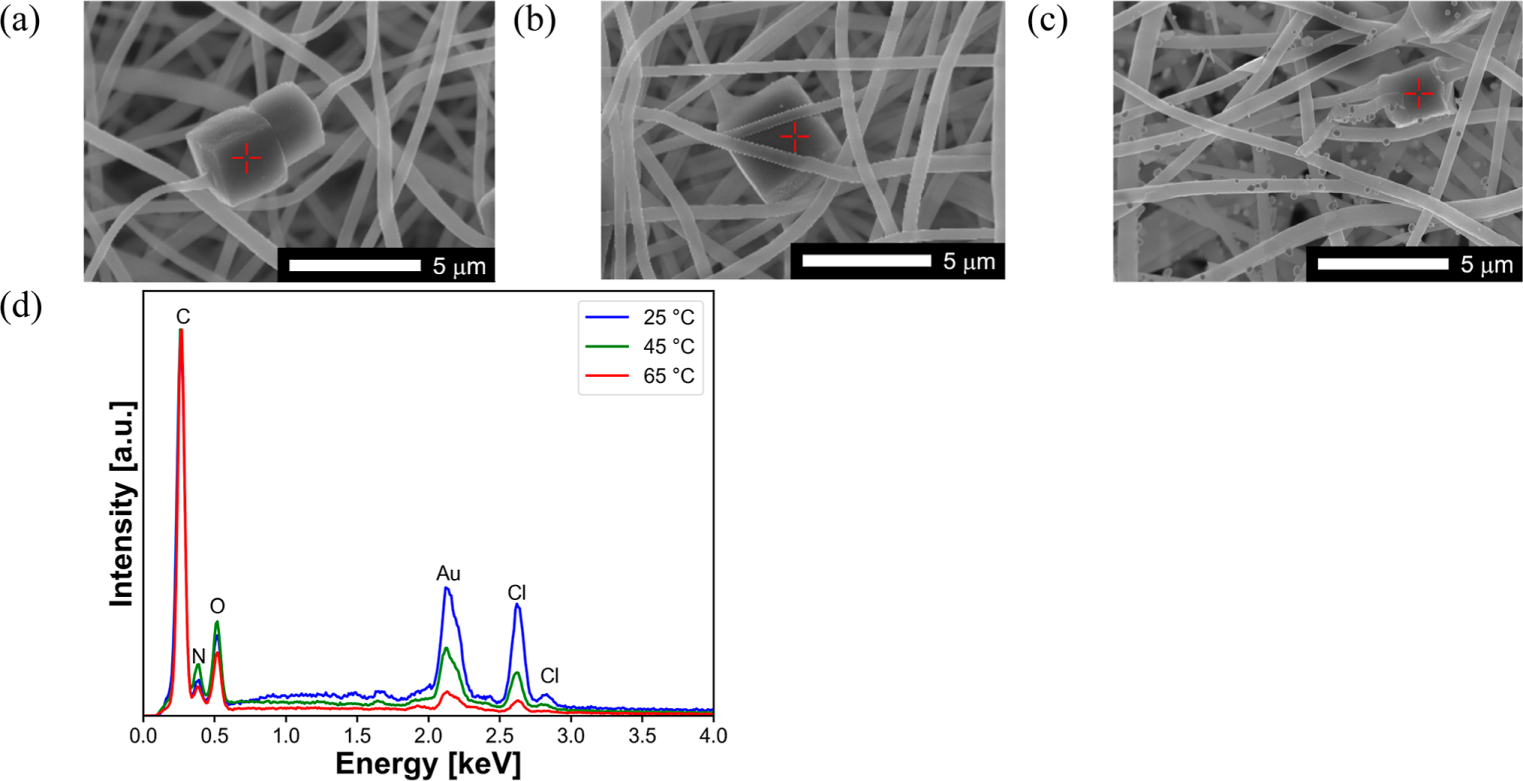
Typical SEM images of the Au-adsorbed PhC@NF web at pH 3 and (a) 25 °C, (b) 45 °C and (c) 65 °C. (d) EDS spot analysis of a single PhC in the web performed at the red spot in (a–c). The intensity was normalized to the peak intensity of carbon atoms.

**Figure 11:**
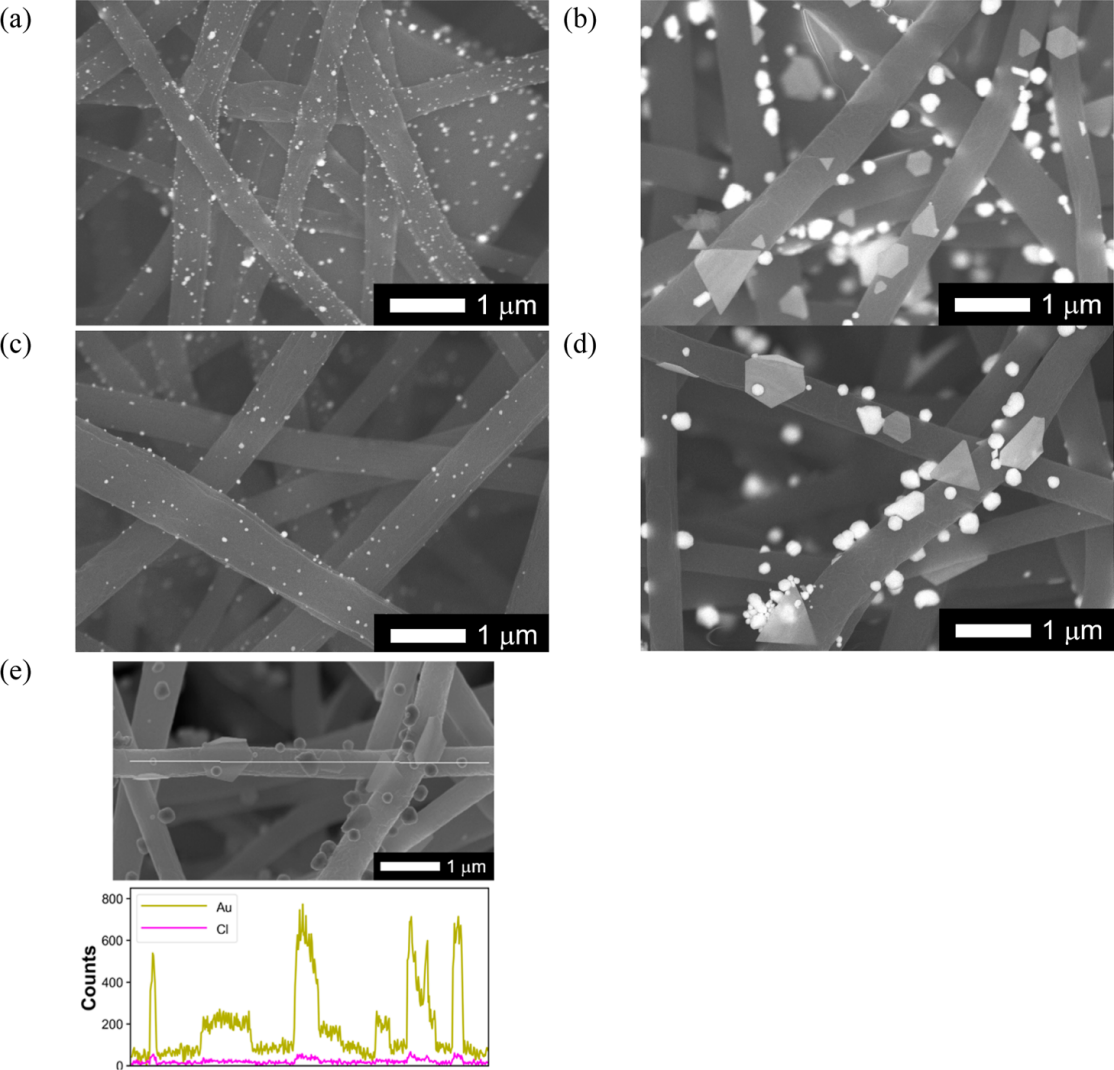
Typical backscattered electron images of the Au-adsorbed PhC@NF web at pH 3 and 45 °C with (a) low Au adsorption capacity (30 mg g^–1^) and (b) high Au absorption capacity (55 mg g^–1^); and the Au-adsorbed PhC@NF web at pH 3 and 65 °C with (c) low Au adsorption capacity (25 mg g^–1^) and (d) high Au absorption capacity (100 mg g^–1^). (e) Typical EDS line analysis along a single NF [the data is for (b)].

This was possibly due to the higher temperature conditions (45 and 65 °C), which promoted the Au­(III) reduction reaction and release of reduced elemental Au from the PhCs. In other words, the PhCs functioned as an “elemental Au generator” via the Au­(III) reduction reaction and subsequent release of Au rather than adsorbed Au within the PhCs. One possibility is that the higher temperature conditions prevent stable confinement of the reduced Au(0) inside the PhCs (i.e., in the above-mentioned Tyr cluster). The released Au was readsorbed onto the EVOH NF surface and grew into NPs and nanoplatelets depending on the nucleation kinetics at the localized NF surface. Therefore, the Au adsorption capacity of the PhC@NF web would be greatly enhanced by the synergetic effect of the intrinsic Au reduction/release ability of the PhCs, the intrinsic Au reduction/adsorption ability of EVOH, and the large surface area of the NFs, reaching the highest Au equilibrium adsorption capacity of 413 mg g^–1^ and almost all of the adsorbed nanostructures were reduced elemental Au. Thus, this innovative protein-nanofiber hybrid material enabled the adsorption of Au ions and their recovery as reduced Au on a membrane.

## Conclusions

In this study, PhC@NF webs were prepared by electrospinning PhCs and EVOH and their Au adsorption behavior was investigated under various pH and temperature conditions. At 25 °C, the PhC@NF web exhibited a *q*_max_ value of 51.7 mg g^–1^ at pH1 and a maximum *K*_L_ value of 1.02 L mg^–1^ at pH 3, which was the highest *K*_L_ value among reported Au ion adsorbents. By contrast, Au reduced by PhCs was deposited as particles on the NF surface at higher temperatures, particularly at 65 °C. The results demonstrated that PhCs are promising biobased materials for efficient Au ion recovery from low-concentration Au ion solutions. To the best of our knowledge, this is the first study to report the unique Au ion adsorption behavior of PhCs. Our DFT calculations suggested that Au ions can interact and/or chemically react with AA residues within PhCs due to their unique coordination environment. However, an in-depth understanding of the adsorption/reduction reactions within and on PhCs is still lacking, particularly at the nano or subnano scale. Further investigations based on precise characterization of Au ion translocation and electronic structure variations via X-ray crystallography, X-ray absorption fine structure analysis, etc., as well as highly accurate theoretical calculations, are warranted.

## Supplementary Material



## Notes

Preparation procedure of PhC@NF webs, Schematic of the electrospinning apparatus, Equilibrium adsorption capacity of various precious metal species on the PhC@NF web at 25 °C and pH 1, Reported Au adsorption performances of representative adsorbents based on Langmuir fitting, Estimation of pH dependence of the fractions of trivalent Au ion species in the adsorbate solution and protonated fraction of basic AA residues within the PhCs, XPS spectra for the purified PhCs before and after Au adsorption, DSC curve of the EVOH NF web without PhCs under an N_2_ atmosphere, Typical surface SEM image of the PhC@NF web after the Au desorption experiment and corresponding EDS mapping of Au-M, Local structures of pristine, [AuCl_3_] coordinated, and [AuCl_2_(OH)] coordinated cystine residues and cystine residue after reduction of [AuCl_2_(OH)­(H_2_O)], TGA curve of purified PhCs under an N_2_ atmosphere., Photograph and backscattered electron image of the Au-adsorbed NF web without PhCs (EVOH NF), and Photographs illustrating the color change of the Au-adsorbed PhC@NF webs (PDF)
