# Supplementary material for: Adsorption Behavior of Gold Ions on Nanofiber Webs Containing Protein Polyhedral Crystals
Source: ACS Omega. 2025 May 27;10(22):23308–19. doi: 10.1021/acsomega.5c01719 (PMC12163754; doi:10.1021/acsomega.5c01719)
Supplement: Supplementary file 1 [file ao5c01719_si_001.pdf]

Supporting Information

Adsorption Behavior of Gold Ions on Nanofiber Webs Containing Protein Polyhedral Crystals

Shuto Matsuura,[†] Takashi Iwahashi,[†] Hajime Mori,[‡,*] Akihiko Tanioka,[†,‡] and Hidetoshi Matsumoto[†,*]

[†]Department of Materials Science and Engineering, Tokyo Institute of Technology, 2-12-1 Ookayama, Meguro-ku, Tokyo, 152-8552, Japan

[‡]Inspired Micro Crystals, 27-1 Shimouchikawara-cho, Koyama, Kita-ku, Kyoto, 603-8132, Japan

*Address correspondence to: matsumoto.h.f6bc@m.isct.ac.jp and silk7776@gmail.com

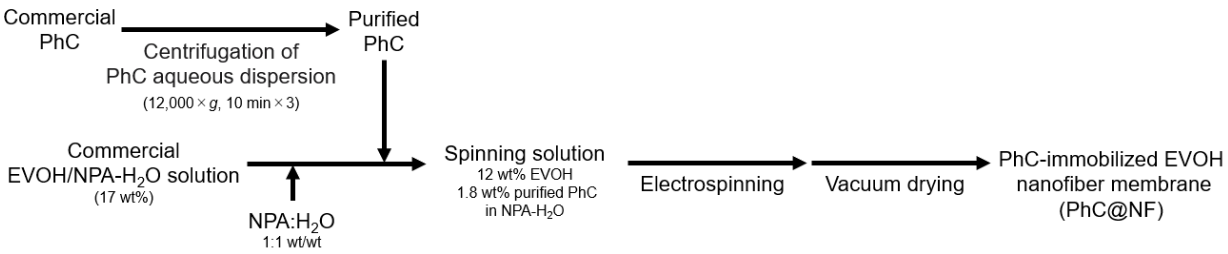

Scheme S1. Preparation procedure of PhC@NF webs.

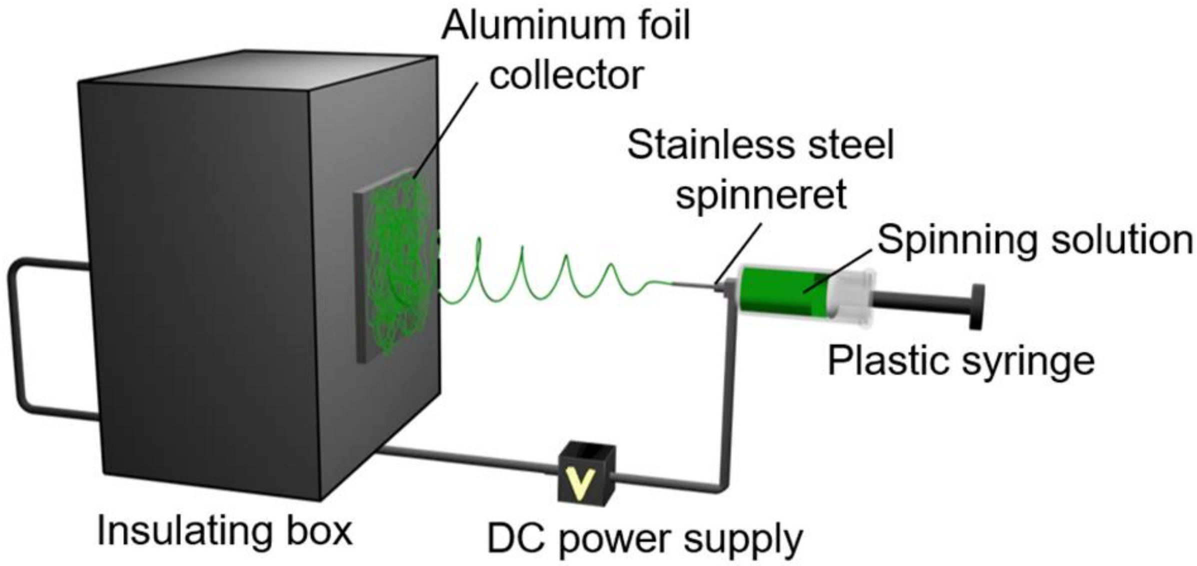

Figure S1. Schematic of the electrospinning apparatus.

Table S1. Compositions of aqueous adsorbent solutions with various Au concentrations and pH values.

Content				
Au [mg·L^{-1}]	$HAuCl_4 \cdot 4H_2O$ [mg·L^{-1}]	HCl [mg·L^{-1}]		
		pH 1	pH 2	pH 3
5	1.05×10^1	3.65×10^3	3.64×10^2	3.55×10^1
10	2.09×10^1	3.64×10^3	3.63×10^2	3.46×10^1
20	4.18×10^1	3.64×10^3	3.61×10^2	3.28×10^1
40	8.36×10^1	3.64×10^3	3.57×10^2	2.91×10^1
60	1.25×10^2	3.63×10^3	3.53×10^2	2.53×10^1
80	1.67×10^2	3.63×10^3	3.50×10^2	2.16×10^1
100	2.09×10^2	3.63×10^3	3.46×10^2	1.79×10^1
150	3.14×10^2	3.62×10^3	3.37×10^2	8.69
200	4.18×10^2	3.61×10^3	3.28×10^2	0
250	5.23×10^2	3.60×10^3	3.18×10^2	—
300	6.27×10^2	3.59×10^3	3.09×10^2	—
1000	2.09×10^3	3.46×10^3	—	—

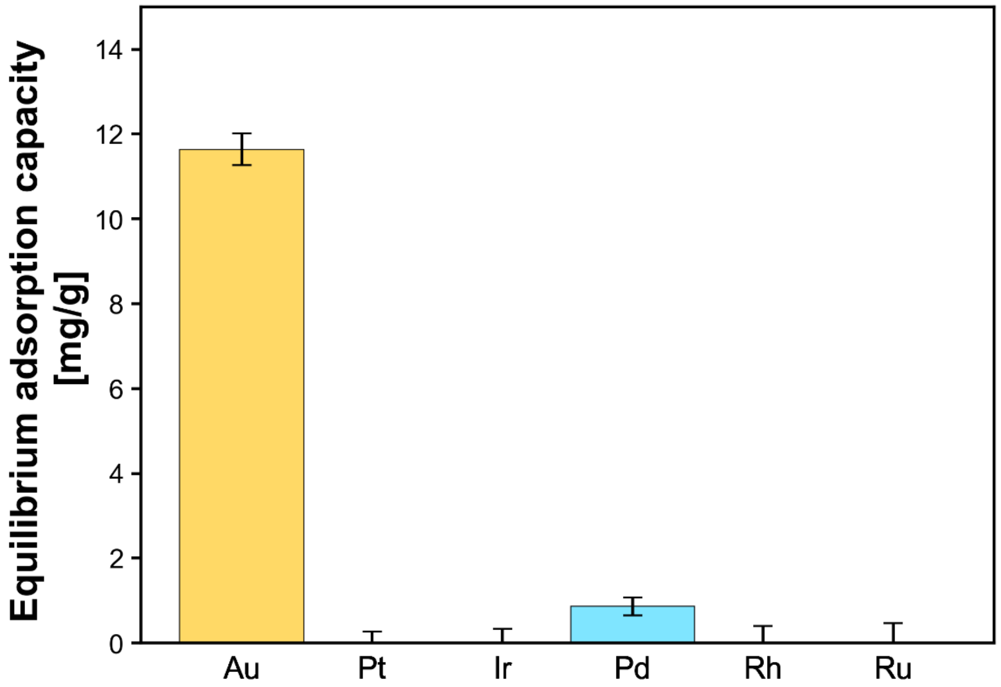

Figure S2. Equilibrium adsorption capacity of various precious metal species on the PhC@NF web at 25°C and pH 1. A mixed adsorbate solution containing 4 mg·L^{-1} of each precious metal was prepared by diluting a commercial standard solution containing Au, Pt, Ir, Pd, Rh, and Ru in 10% hydrochloric acid in ultrapure water (100 μg·mL^{-1}), Calib. Std #2 Precious Metals (AccuStandard, New Haven, CT, USA). Three samples were measured for each metal, and the mean value (±standard deviation) is indicated.

Table S2. Reported Au adsorption performances of representative adsorbents based on Langmuir fitting.

Adsorbent	Solution pH	Temperature [°C]	q_{max} [mg·g^{-1}]	K_L [L·mg^{-1}]	R^2	Reference
**PhC@NF web**	1	25	51.7	0.00544	0.976	**This Work**
	2	25	38.4	0.167	0.961	
	3	25	27.8	1.02	0.903	
	3	45	54.1	11.3	0.995	
	3	65	368	0.0945	0.960	
PTL bilayer membrane	3	10	758	0.202	0.936	[1]
	3	37	777	0.261	0.886	
	3	60	1034	1.12	0.978	
Microporous polyurea	2	25	1301	0.218	1.00	[2]
Cr-based MOF	1.65	25	357	0.337	0.995	[3]
Core–shell Fe_3O_4@CuS	6	25	559	5.4×10^{-6}	0.997	[4]
$Ti_3C_2T_x$ nanosheets	7	25	1952	0.102	1.00	[5]
Amberjet 4200	5	25	164	0.173	0.999	[6]

Abbreviations: PTL, phase-transitioned lysozyme; Amberjet 4200, Styrene-*co*-divinylbenzene based anion exchange resin produced by Rohm and Haas.

Estimation of pH dependence of the fractions of trivalent Au ion species in the adsorbate solution and protonated fraction of basic AA residues within the PhCs

The fractions of Au species were estimated by considering the following hydrolysis reactions:

$$AuCl_4^- + H_2O \rightleftarrows AuCl_3(H_2O) + Cl^- \tag{S1}$$

$$AuCl_3(H_2O) \rightleftarrows AuCl_3(OH)^- + H^+ \tag{S2}$$

$$AuCl_3(OH)^- + H_2O \rightleftarrows AuCl_2(H_2O)(OH) + Cl^- \tag{S3}$$

$$AuCl_2(H_2O)(OH) + H_2O \rightleftarrows AuCl_2(OH)_2^- + H^+ \tag{S4}$$

$$AuCl_2(OH)_2^- + H_2O \rightleftarrows AuCl(OH)_3^- + Cl^- + H^+ \tag{S5}$$

$$AuCl(OH)_3^- + H_2O \rightleftarrows Au(OH)_4^- + Cl^- + H^+ \tag{S6}$$

The relative proportion of the products to the reactants for each reaction was calculated using the law of mass action. The following equation represents the law of mass action for **Formula S6**.

$$K = \frac{[\mathrm{Au(OH)_4}^-][\mathrm{Cl}^-][\mathrm{H}^+]}{[\mathrm{AuCl(OH)_3}^-][\mathrm{H_2O}]} \tag{S7}$$

where K is the equilibrium constant[7], $[H_2O]$ is 55.6 M, $[H^+]$ is determined as a function of pH and $[Cl^-]$ is equal to $[H^+]$ in this system.

The desired fractions were obtained by simultaneously solving all of the determined equilibrium constants (**Formulas S1–S6**).

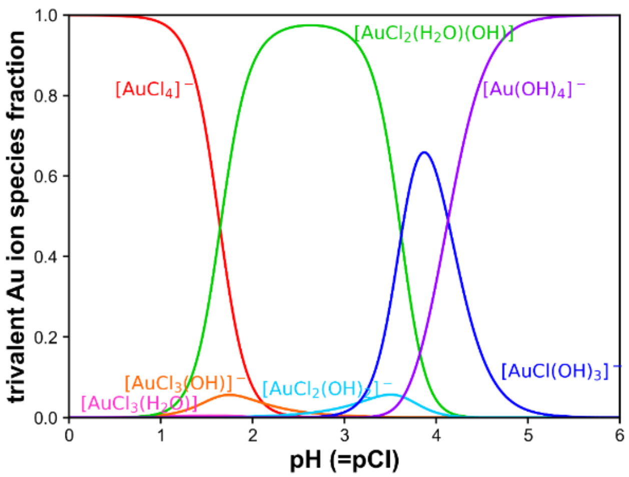

Figure S3. The estimated pH dependence of the fractions of trivalent Au ion species in the adsorbate solution.

Table S3. The p*K*a values of side chains of ionizable AAs within the PhCs.[8]

amino acids	p*K*a
Aspartic acid (Asp)	3.65
Glutamic acid (Glu)	4.25
Histidine (His)	6.00
Cystine (Cys)	8.18
Tyrosine (Tyr)	10.07
Lysine (Lys)	10.53
Arginine (Arg)	12.48

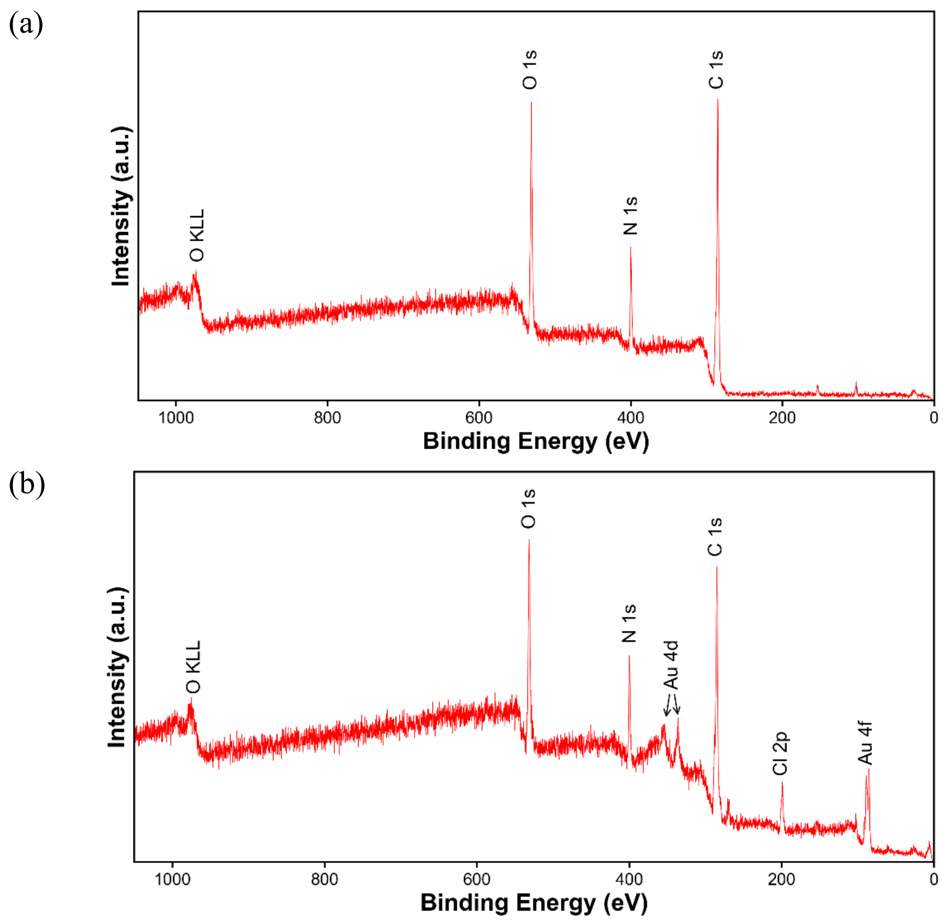

Figure S4. XPS spectra for the purified PhCs (a) before and (b) after Au adsorption (adsorption quantity = 300 mg·g-PhC^{-1} at 25°C and pH 1).

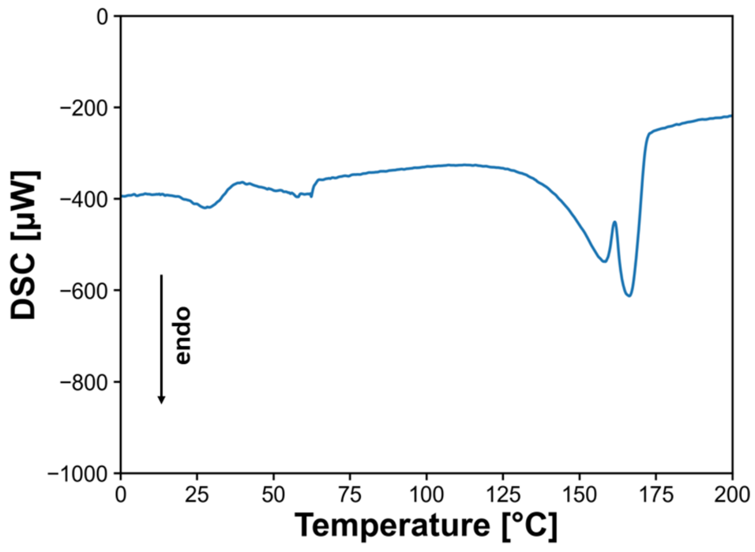

Figure S5. DSC curve of the EVOH NF web without PhCs under an N_2 atmosphere.

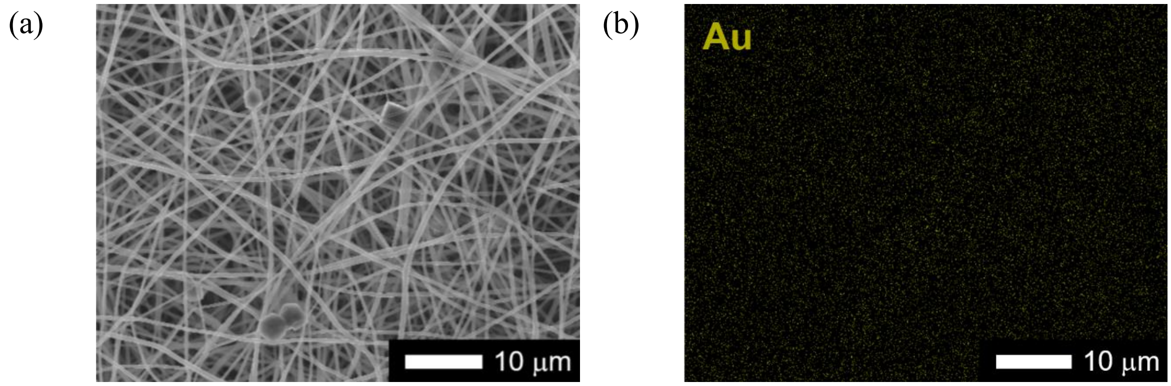

Figure S6. (a) Typical surface SEM image of the PhC@NF web after the Au desorption experiment and (b) corresponding EDS mapping of Au-M.

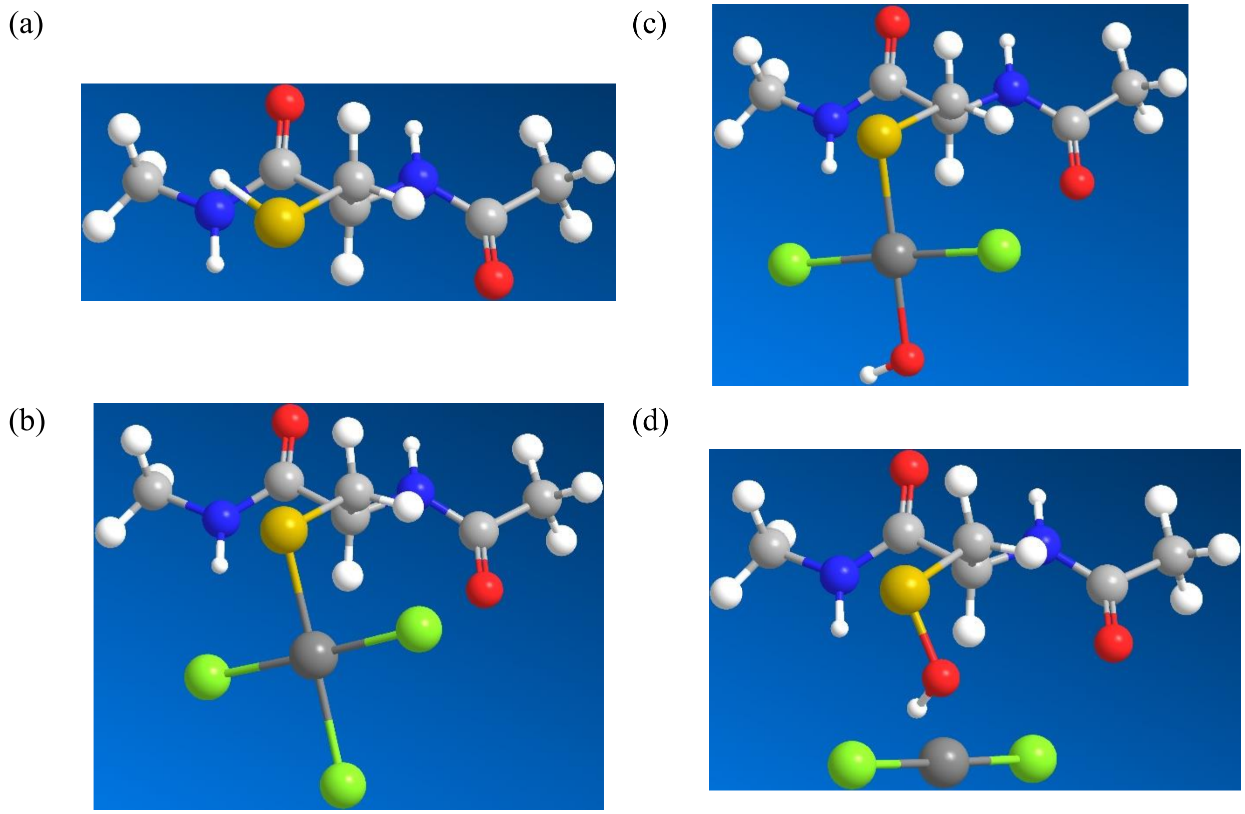

Figure S7. Local structures of (a) pristine, (b) [$AuCl_3$] coordinated, and (c) [$AuCl_2(OH)$] coordinated cystine residues, and (d) cystine residue after reduction of [$AuCl_2(OH)(H_2O)$]. White: hydrogen atoms; gray: carbon atoms; blue: nitrogen atoms; red: oxygen atoms; yellow: sulfur atoms; green: chlorine atoms; and dark gray: gold atoms.

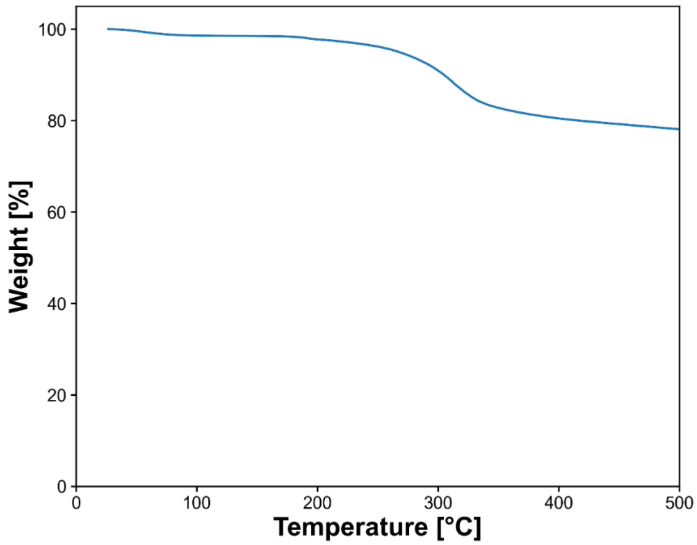

Figure S8. TGA curve of purified PhCs under an N_2 atmosphere.

(a)

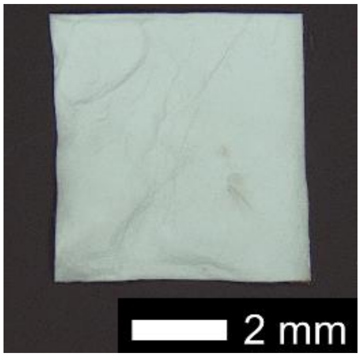

(b)

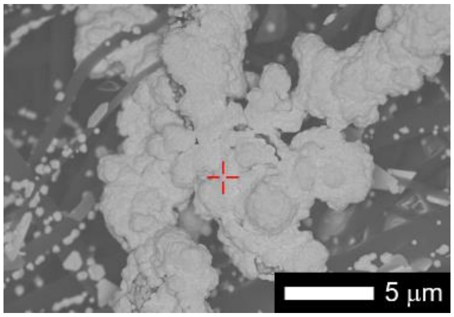

(c)

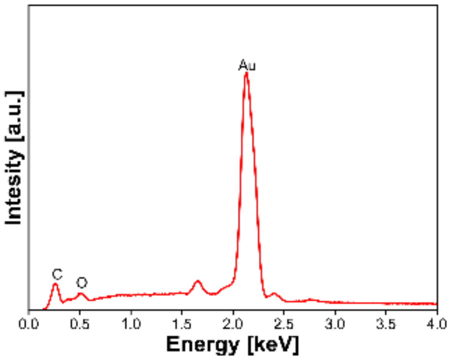

Figure S9. (a) Photograph (off-white) and (b) backscattered electron image of the Au-adsorbed NF web without PhCs (EVOH NF) with an adsorption capacity of 140 mg·g^{-1} at 65°C and pH 3. (c) EDS analysis performed at the red spot in (b).

(a) (b) (c) (d) (e) (f)

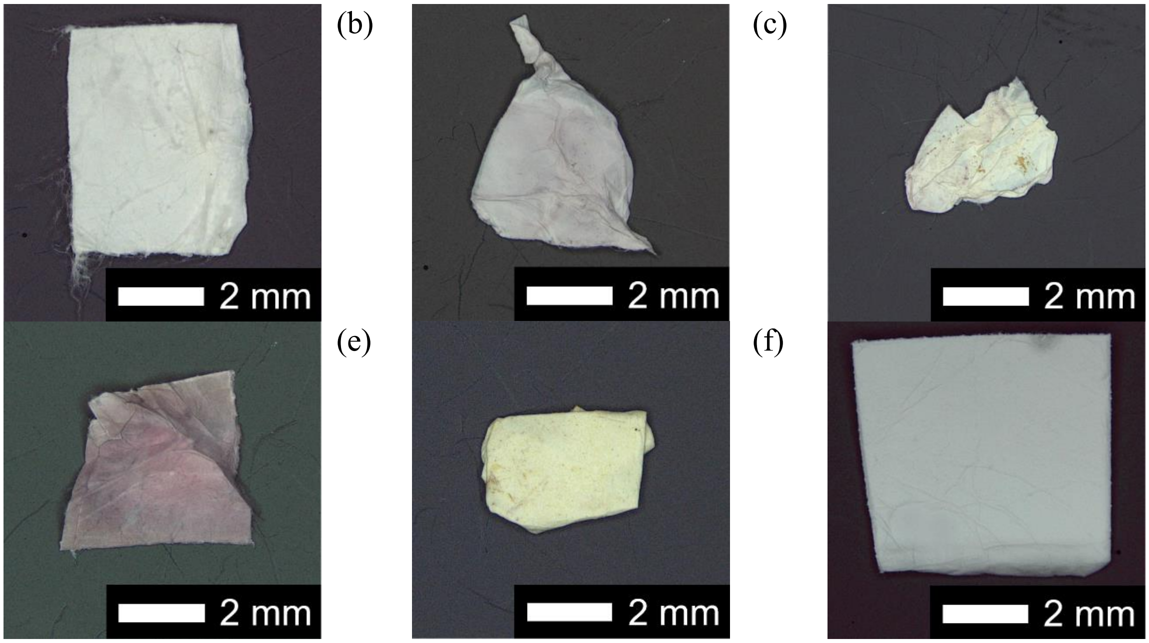

Figure S10. Photographs illustrating the color change of the Au-adsorbed PhC@NF webs with Au adsorption capacities of (a) 30 mg·g^{-1} at 25°C (off-white), (b) 30 mg·g^{-1} at 45°C (pale pink), (c) 55 mg·g^{-1} at 45°C (pale yellow), (d) 30 mg·g^{-1} at 65°C (pink), and (e) 100 mg·g^{-1} at 65°C (yellow). All adsorption experiments were performed at pH 3. (f) The PhC@NF web before Au adsorption for comparison (white).

REFERENCES

1. Yang, F.; Yan, Z.; Zhao, J.; Miao, S.; Wang, D.; Yang, P. Rapid Capture of Trace Precious Metals by Amyloid-like Protein Membrane with High Adsorption Capacity and Selectivity. *J. Mater. Chem. A* **2020**, *8*(6), 3438–3449. https://doi.org/10.1039/c9ta12124b.
2. Lin, B.; Chen, W.; Lei, Y.; Ma, X.; Wang, J.; Li, L. Solvothermal Preparation of Microporous Polyureas for Au(III) Adsorption. *Langmuir* **2024**, *40*(17), 9001–9011. https://doi.org/10.1021/acs.langmuir.4c00305.
3. Wang, B.; Ma, Y.; Xu, W.; Tang, K. Cr-Based MOF for Efficient Adsorption of Au at Low Concentrations. *Langmuir* **2022**, *38*(29), 8954–8963. https://doi.org/10.1021/acs.langmuir.2c01137.
4. Xia, J.; Ghahreman, A. Core–Shell Structured Fe_3O_4@CuS for Effective Gold Capture and Recovery. *ACS Appl. Nano Mater.* **2023**, *6*(12), 10837–10844. https://doi.org/10.1021/acsanm.3c01772.
5. Liu, X.; Ma, L.; Han, P.-W.; Yang, Z.; Li, Z.; Yan, J.; Wang, Y.; Ye, S. Ultrahigh Capacity and Rapid Selective Recycling of Gold Ions by Organic Intercalated and Exfoliated Few-Layer $Ti_3c_2T_x$ Nanosheets. *ACS Sustainable Chem. Eng.* **2022**, *10*(46), 15305–15318. https://doi.org/10.1021/acssuschemeng.2c05143.
6. Choi, J.-W.; Song, M.-H.; Bediako, J. K.; Yun, Y.-S. Sequential Recovery of Gold and Copper from Bioleached Wastewater Using Ion Exchange Resins. *Environ. Pollut.* **2020**, *266*(3), 115167. https://doi.org/10.1016/j.envpol.2020.115167.
7. Nechayev, Y. A.; Zvonareva, G. V. Adsorption of Gold(III) Chloride Complexes on Hematite (in Russian). *Geokhimiya* **1983**, *6*, 919–924.
8. Lide, D. R. *CRC Handbook of Chemistry and Physics: A Ready-Reference Book of Chemical*

and Physical Data; CRC Press: Boca Raton, Fl, 1992.
